# Endovascular treatment of left internal thoracic artery aneurysm

**DOI:** 10.1590/1677-5449.200042

**Published:** 2020-08-31

**Authors:** Milton Sérgio Bohatch, Tércio Tanure, André Luiz de Oliveira, Maurício Serra Ribeiro, Edwaldo Edner Joviliano

**Affiliations:** 1 Universidade de São Paulo – USP, Faculdade de Medicina de Ribeirão Preto, Hospital das Clínicas, Divisão de Cirurgia Vascular e Endovascular, Ribeirão Preto, SP, Brasil.

**Keywords:** aneurism, mammary arteries, endovascular procedures

## Abstract

Aneurysm of the internal thoracic artery is a rare entity, with variable presentation and a potential risk of fatal rupture. Angiotomography is the diagnostic test of choice and is useful for planning treatment. Considering the morbidity of thoracic access for a direct approach and the unpredictable risk of rupture, an endovascular procedure is the treatment modality of choice for this type of aneurysm. We describe the case of an internal thoracic artery aneurysm discovered incidentally during investigation of syncope and treated by embolization with low-profile and controlled-release microcoils.

## INTRODUCTION

Internal thoracic artery aneurysms (ITAAs) are rare entities, generally found as pseudoaneurysms after sternotomy, endovascular procedures, or thoracic traumas.^1^ The first case was described in 1973 by Martin et al.^2^ after wiring closed a sternotomy. Just 40 cases have been described over the last 40 years, two thirds of which were pseudoaneurysms.^3^ True aneurysms are rarer and the first case was reported in 1978 by Den Otter and Stam in a 30-year-old woman with a “coin lesion” found incidentally during a routine X-ray examination.^4^ True aneurysms have been described in association with vasculitis, connective tissue disorders, genetic syndromes, and atherosclerosis.^5^ Presentation of ITAAs can be variable, with findings such as anterior mediastinal mass, hemothorax, or hemoptysis, but they may also be asymptomatic and found incidentally.^6^ While ITAAs are small, rupture can be fatal and the most common initial manifestation is hemothorax with hypovolemic shock.^7^ Diagnosis is generally founded on the classic “coin lesion” finding seen on simple chest X-rays or on presence of a mass in the anterior mediastinum observed on computed tomography of the thorax.^8^ Angiotomography can be used to study the aneurysm in detail, which is important for planning treatment.^6^ The treatment options for pseudoaneurysms and true aneurysms are the same.^4^ Minimally invasive treatment using endovascular techniques with coil embolization or stenting has become the first choice option for treatment of smaller aneurysms.^9^ We describe a rare case of ITAA, discovered incidentally during investigation of syncope and treated with coil embolization.

## CASE DESCRIPTION

**Figure 1:**
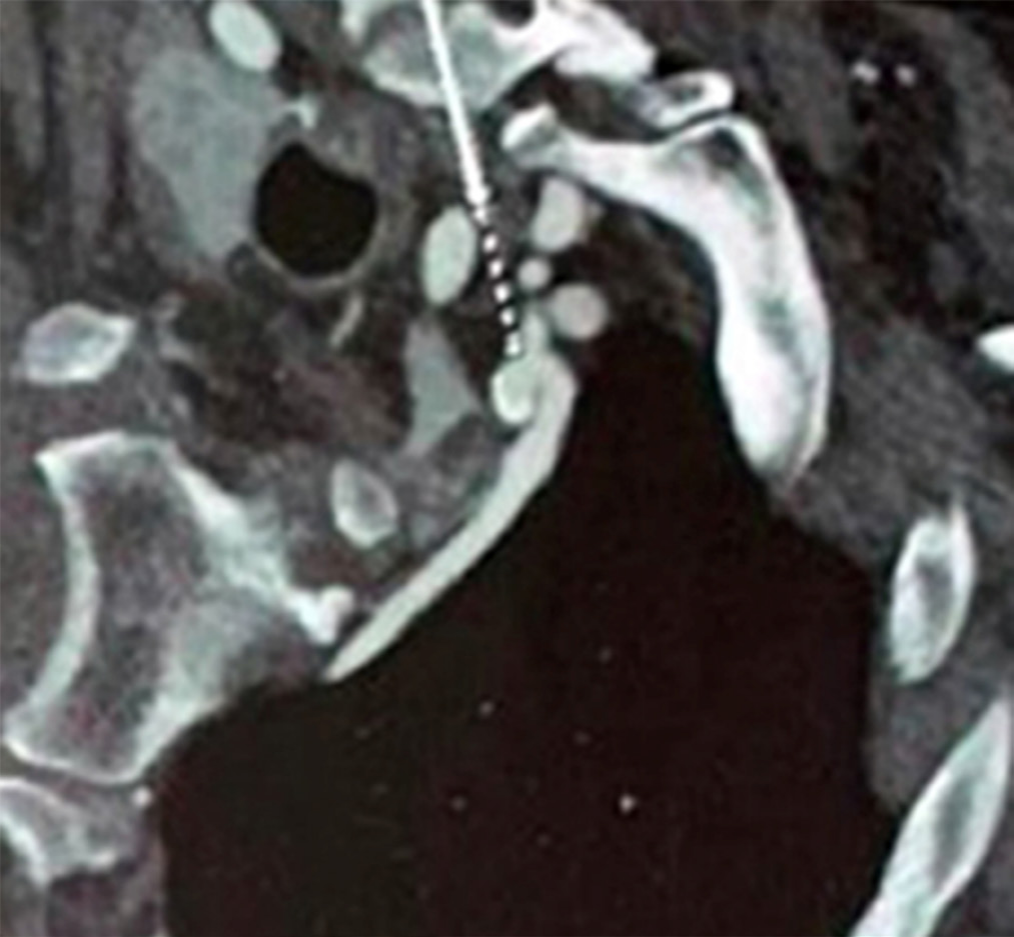
Angiotomography showing a saccular aneurysm of the left internal thoracic artery, with parietal calcification and without contrast leakage (white arrow).

The patient was a 63-year-old female, with a history of diabetes and arterial hypertension, but no prior thoracic surgery or traumas and no symptoms of intermittent claudication or cerebrovascular disease. She underwent coronary angiotomography to investigate episodes of syncope, with an incidental finding of a saccular aneurysm of the left internal thoracic artery. Her vascular physical examination did not detect any murmurs or thrills in the carotid, abdominal aorta, or femoral regions and distal pulses were present and symmetrical. Angiotomography showed a saccular aneurysm in the proximal third of the left internal thoracic artery, about 5 mm from its ostium, with a largest diameter of 9.5 mm (Figure 1), and signs of atherosclerotic disease involving the coronary arteries, with significant stenosis of the proximal third of the anterior descending artery. Although the aneurysm diameter was still less than 1 cm, the decision was taken to treat because of the unpredictable risk of rupture in a relatively young patient.

**Figure 2:**
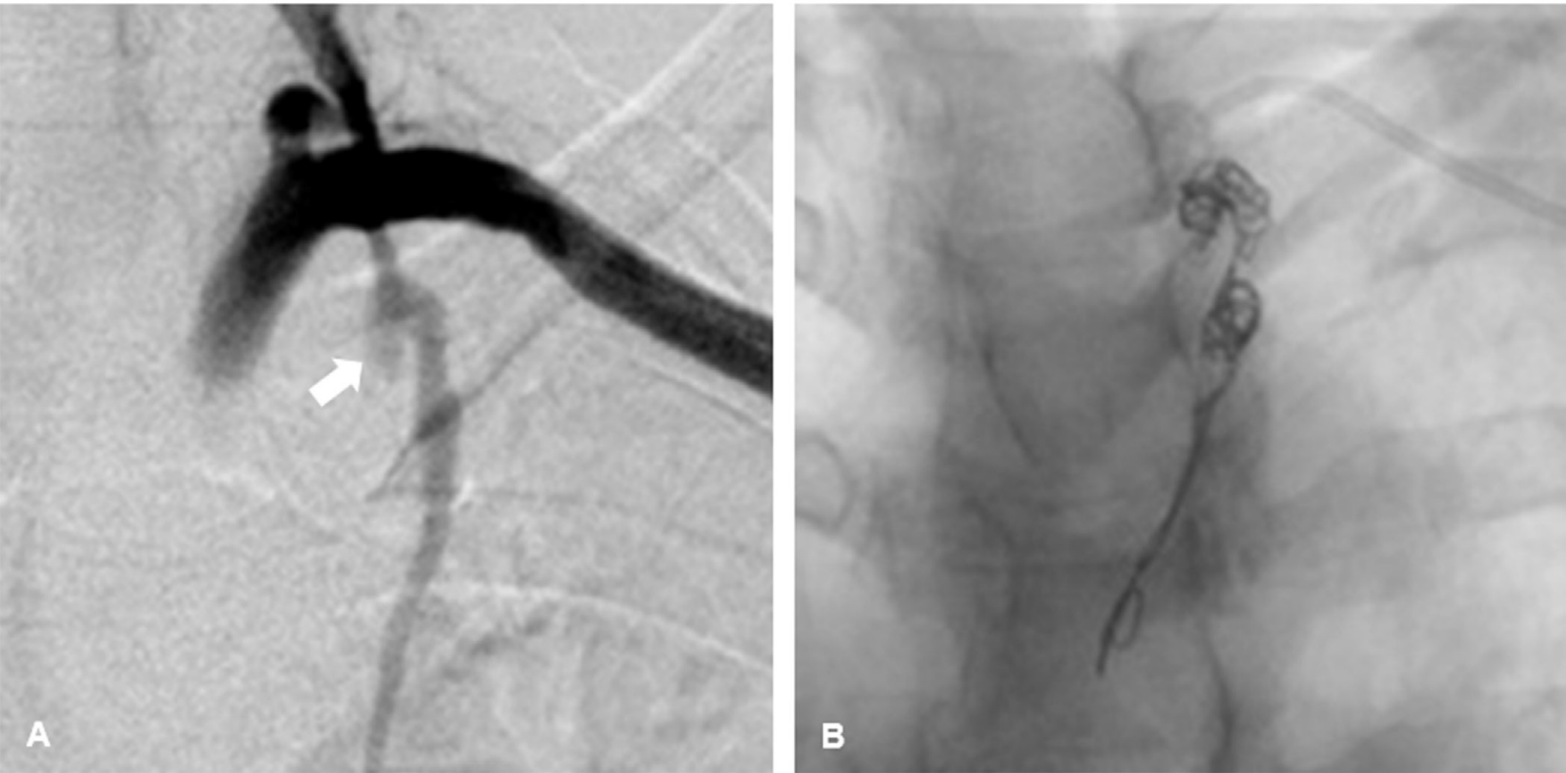
Angiography showing saccular aneurysm of the left internal thoracic artery without leakage of contrast. (A) Oblique view (white arrow); (B) Embolization with 10 mm x 30 cm and 12 mm x 30 cm controlled-release coils, (Concerto^®^; Medtronic®, Minneapolis, United States).

The patient underwent endovascular treatment with access via puncture of the left brachial artery with a 6 Fr introducer, followed by superselective catheterization of the internal thoracic artery, using a 5 Fr vertebral catheter followed by a 2.7 Fr microcatheter. Angiography showed the saccular aneurysm soon after the origin of the left internal thoracic artery, with a diameter of around 1 cm and no signs of contrast leakage (Figure 2A). The ITAA was occluded using two controlled-release coils, one 10 mm x 30 cm and the other 12 mm x 30 cm, (Concerto^®^, Medtronic, Minneapolis, United States), extending from the distal segment to the proximal segment of the aneurysm (Figure 2B). Control arteriography showed that the subclavian artery was patent and that contrast was not filling the aneurysm sac in the internal thoracic artery. There were no transoperative complications and the patient was discharged from hospital on the first day after the operation. The patient gave her consent for this case report, including publication of the images.

## DISCUSSION

The internal thoracic artery emerges from the first portion of the subclavian artery and immediately descends close to the pleura in the upper intercostal space. At the sixth intercostal space, it divides into the superior epigastric artery and the musculophrenic artery. It is responsible for supplying blood to the anterior chest wall and the breasts.^9^^,^^10^ The mean diameter of this artery is small (around 2 mm), but its flow rate can reach 150 mL/min, and it can cause severe and even fatal bleeding.^11^^,^^12^

The etiology of true aneurysms of the internal thoracic artery is generally related to vasculitis (Kawasaki disease, polyarteritis nodosa, and systemic lupus erythematosus), connective tissue diseases (Marfan Syndrome and Ehlers-Danlos Syndrome), type 1 neurofibromatosis, fibromuscular dysplasia, atherosclerosis, or idiopathic causes.^3^^,^^7^ Although endovascular treatment does not offer the possibility of definitive diagnosis by histopathology, it is presumed that the diagnosis in this case was a true atherosclerotic aneurysm, based on the patient’s clinical history of hypertension and diabetes, signs of atherosclerotic disease with calcifications of coronary arteries, negative history of prior medical interventions or traumas, and no diagnosis of connective tissue diseases or vasculitis, and also on the findings of examinations. These elements lead us to assume that the aneurysm was a true aneurysm of atherosclerotic degenerative origins. Histopathological analysis of aneurysms shows that atherosclerotic degeneration is the major cause, but there are reports of degeneration of the tunica media and fibromuscular dysplasia associated with their occurrence.^13^

The characteristics that indicate risk with ITAAs are rapid growth and high risk of rupture.^1^ It is not uncommon that they are detected as incidental findings in radiological examinations even after previous negative examinations.^1^ In several cases, the only symptom presented was progressive chest pain. There are also reports of dyspnea, continuous murmur, thoracic thrill, painful parasternal edema, and even supraclavicular or intercostal masses. Around 37% of cases manifest with aneurysm rupture, causing massive hemothorax and potential risk of death.^1^ The lethality of these aneurysms is because of their location within the thoracic cavity. The subatmospheric intrathoracic pressure, the dynamic movement of the chest wall, and the relative lack of adjacent supportive tissue create an ideal environment for the aneurysm to grow and for massive bleeding if it ruptures.^11^ Additionally, expansion of the aneurysm or contained hematoma can lead to compression and paralysis of the phrenic nerve.^11^ These are the main reasons for indicating surgical treatment of ITAAs.^1^

The exact description of the size and anatomic site of the ITAA is crucial for planning surgery. Of the many different noninvasive examinations available, angiography by multislice tomography is the imaging exam of choice for diagnosis. This examination can show the aneurysm in great detail, using post-processing techniques with multiplanar formatting and volume rendering.^11^ The aneurysm wall is generally smooth and well-defined, with the exception of mycotic aneurysms, which can have a thicker, irregular, and poorly-defined wall. Multislice tomography angiography can also show the feeder vessel and collateral vessels, which are important for planning surgery.^14^

True ITAAs are so rare that there is little information on management and prognosis. To date, there are no established criteria for intervention^3^ and the decision to treat ITAAs is based on their size, on the presence of symptoms, and on the risk of rupture.^8^ Internal thoracic artery aneurysm rupture can be fatal, since hemothorax with shock is the most common initial manifestation.^3^ Treatment options described in the literature are open surgical repair or endovascular treatment with stenting and/or embolization. In the case of rupture, treatment depends on the patient’s hemodynamic condition.

In patients who are unstable after rupture, open surgery is still considered the method of choice.^1^ Surgical exploration includes removal of the hematoma, surgical ligature of the vessel, and packing to achieve hemostasis.^15^ However, surgery is aggressive and it can be difficult to identify the source of bleeding, while surgical ligature can be complicated by the fragility of the vascular tissue. Miura et al.^16^ reported a frustrated attempt to identify the cause of bleeding in a patient with a ruptured aneurysm of the intercostal artery. Surgical repair involves other risks, such as bleeding, infection of the surgical site, injury to adjacent structures, risks related to anesthesia, slow recovery, and extended length of hospital stay.^8^^,^^14^ As a result, endovascular treatment is being adopted as an effective treatment option that is safe and less invasive, even for hemodynamically unstable patients.^17^

In patients who are hemodynamically stable, endovascular treatment is the first line option, because it is a minimally invasive technique widely used in the elderly, critical patients, those with coagulation disorders, and in cases with special conditions, such as patients with Marfan and Loeys-Dietz syndromes.^11^^,^^13^ Embolization with coils is the treatment of choice for arteriovenous fistulas and smaller aneurysms because the technique is relatively easy in tortuous vessels and cases with short aneurysm necks.^3^ There are also other agents used for embolization, such as polymers and sometimes even glues, that can be used in combination treatment with the objective of occluding the proximal and distal portions of the aneurysm to avoid it being fed by collaterals.^11^

Exclusion of the aneurysm using covered stents is an option for certain vascular beds and some studies have shown that this is a feasible alternative treatment for ITAA.^18^ Alhawasli et al.^19^ reported successful bilateral exclusion of ITAAs employing sequential covered stents in a patient with Marfan Syndrome. Some authors believe that it is beneficial to preserve patency of the internal thoracic artery, particularly in patients with high cardiovascular risk, taking into consideration the possibility of a future need to conduct myocardial revascularization.^1^

Complications related to endovascular procedures that have been identified include reflux via collaterals causing expansion of the aneurysm and recurrence of bleeding after embolization.^11^ However, the success rate of embolized ITAAs is 94.3%.^11^ Endovascular techniques therefore constitute a minimally invasive, safe, and effective option and are currently the treatment of choice for ITAAs.^14^
