# Supplementary material for: Endovascular treatment of left internal thoracic artery aneurysm
Source: J Vasc Bras. 2020 Aug 31;19:e20200042. doi: 10.1590/1677-5449.200042 (PMC8202176; doi:10.1590/1677-5449.200042)
Supplement: Translation

# Translation: Tratamento endovascular de aneurisma de artéria torácica interna esquerda

**Authors:** Milton Sérgio Bohatch, Tércio Tanure, André Luiz de Oliveira, Maurício Serra Ribeiro, Edwaldo Edner Joviliano

**Affiliations:** 1 Universidade de São Paulo – USP, Faculdade de Medicina de Ribeirão Preto, Hospital das Clínicas, Divisão de Cirurgia Vascular e Endovascular, Ribeirão Preto, SP, Brasil.

**Keywords:** aneurisma, artéria torácica interna, procedimentos endovasculares

## Abstract

O aneurisma da artéria torácica interna é uma entidade rara, com apresentação variável e risco potencial de ruptura e de morte. A angiotomografia é o exame diagnóstico de escolha, sendo útil para o planejamento terapêutico. Considerando morbidade do acesso torácico para abordagem direta e o risco imprevisível de ruptura, o procedimento endovascular se apresenta como modalidade terapêutica de escolha para tratamento desse tipo de aneurisma. Descrevemos um caso de aneurisma de artéria torácica interna, com descoberta incidental na investigação de síncope tratado com embolização com micromolas de baixo perfil e de liberação controlada.

## INTRODUÇÃO

Os aneurismas da artéria torácica interna (AATIs) são entidades raras, relatadas principalmente como pseudoaneurismas após esternotomia, procedimentos endovasculares ou após trauma torácico^1^. O primeiro caso foi descrito em 1973 por Martin et al.^2^ após esternorrafia. Nos últimos 40 anos, apenas quarenta casos foram descritos, sendo dois terços compostos por pseudoaneurismas^3^. Os aneurismas verdadeiros são mais raros, com o primeiro caso reportado em 1978 por Den Otter e Stam em uma mulher de 30 anos com achado incidental de “lesão em moeda” em exame radiológico de rotina^4^. Os aneurismas verdadeiros têm sido descritos em associação com vasculites, distúrbios do tecido conjuntivo, síndromes genéticas e aterosclerose^5^. Os AATIs podem ter apresentações variáveis como massa mediastinal anterior, hemotórax, hemoptise ou, ainda, podem ser assintomáticos encontrados incidentalmente^6^. Embora os AATIs sejam pequenos, a ruptura desses aneurismas pode ser fatal, sendo o hemotórax com choque hipovolêmico a manifestação inicial mais comum^7^. O diagnóstico é geralmente baseado no achado clássico de “lesão em moeda” na radiografia simples de tórax ou na presença de massa no mediastino anterior observada na tomografia computadorizada de tórax^8^. A angiotomografia possibilita o estudo detalhado do aneurisma, importante para o planejamento terapêutico^6^. As opções de tratamento para pseudoaneurismas e aneurismas verdadeiros são as mesmas^4^. O tratamento minimamente invasivo, através de técnicas endovasculares com embolização por molas ou implante de stents, tornou-se a opção de escolha para o tratamento de aneurismas menores^9^. Descrevemos o caso raro de AATI com descoberta incidental na investigação de síncope, tratado com embolização por molas.

## DESCRIÇÃO DO CASO

Paciente de sexo feminino, 63 anos, com antecedentes de diabetes e hipertensão arterial, sem passado cirúrgico torácico ou de traumas e sem sintomas de claudicação intermitente ou doença cerebrovascular. Ela foi submetida a angiotomografia coronariana para investigação de episódios de síncope, com achado incidental de aneurisma sacular de artéria torácica interna esquerda. O exame físico vascular não demonstrou sopros ou frêmitos em território carotídeo, de aorta abdominal ou em femorais e os pulsos distais eram presentes e simétricos. A angiotomografia evidenciou aneurisma sacular no terço proximal da artéria torácica interna esquerda, a cerca de 5 mm do seu óstio, medindo 9,5 mm no maior diâmetro (Figura 1), e sinais de doença aterosclerótica em coronárias com estenose significativa no terço proximal da descendente anterior. Apesar de diâmetro ainda menor que 1 cm, mas com risco imprevisível de ruptura em paciente relativamente jovem, optou-se pela realização do tratamento.

**Figura 1:** Angiotomografia demonstrando aneurisma de artéria torácica interna esquerda, sacular, com calcificação parietal e sem extravasamento de contraste (seta branca).

A paciente foi submetida ao tratamento endovascular com acesso por punção em artéria braquial esquerda com introdutor 6 *Fr*, seguido pelo cateterismo superseletivo da artéria torácica interna, utilizando um cateter vertebral 5 *Fr* e, posteriormente, microcateter 2.7 *Fr*. A angiografia revelou o aneurisma sacular logo após a origem da artéria torácica interna esquerda, com cerca de 1 cm, sem sinais de extravasamento de contraste (Figura 2A). O AATI foi ocluído com duas molas de liberação controlada, 10 mm x 30 cm e 12 mm x 30 cm (Concerto^Ⓡ^, Medtronic, Minneapolis, EUA), que foram alocadas abrangendo desde o segmento distal até o segmento proximal ao aneurisma (Figura 2B). A arteriografia de controle demonstrou perviedade da artéria subclávia e ausência de preenchimento por contraste do saco aneurismático na artéria torácica interna. Não ocorreram complicações transoperatórias e a paciente recebeu alta hospitalar no primeiro pós-operatório. A paciente consentiu em ter seu caso relatado e suas imagens divulgadas.

**Figura 2:** Angiografia demonstrando aneurisma de artéria torácica interna esquerda, sacular, sem extravasamento de contraste. (A) Incidência oblíqua (seta branca); (B) Embolização com molas de liberação controlada, 10 mm x 30 cm e 12 mm x 30 cm (Concerto^Ⓡ^; Medtronic^Ⓡ^, Minneapolis, EUA).

## DISCUSSÃO

A artéria torácica interna surge da primeira porção da artéria subclávia e passa imediatamente para baixo perto da pleura no espaço intercostal superior. No sexto espaço intercostal, divide-se em artéria epigástrica superior e musculofrênica. É responsável pelo suprimento sanguíneo da parede torácica anterior e para as mamas^9^^,^^10^. O diâmetro médio da artéria é pequeno (cerca de 2 mm), mas sua taxa de fluxo pode chegar a 150 mL/min, podendo causar sangramento grave e até fatal^11^^,^^12^.

A etiologia dos aneurismas verdadeiros de torácica interna, em geral, está relacionada a vasculites (doença de Kawasaki, poliarterite nodosa e lúpus eritematoso sistêmico), doenças do tecido conjuntivo (síndrome de Marfan e síndrome de Ehlers-Danlos), neurofibromatose tipo 1, displasia fibromuscular, aterosclerose e causa idiopática^3^^,^^7^. Embora o tratamento endovascular não permita realizar o diagnóstico definitivo pelo exame anatomopatológico, presume-se o diagnóstico de aneurisma verdadeiro aterosclerótico para este caso com base na história clínica — paciente com antecedentes de hipertensão e diabetes, sinais de doença aterosclerótica com calcificações em coronárias, história negativa de intervenções médicas ou de trauma e sem diagnóstico de doenças do tecido conjuntivo ou vasculites — e nos achados de exames. Esses aspectos nos levaram a considerar esse aneurisma como verdadeiro e de causa degenerativa aterosclerótica. A análise histopatológica dos aneurismas revela a degeneração aterosclerótica como a principal causa, mas há relatos de degeneração da túnica média e displasia fibromuscular associadas à sua ocorrência^13^.

As características de risco dos AATIs são o crescimento rápido e o alto risco de ruptura^1^. Não são incomuns achados incidentais em exames radiológicos mesmo após exames anteriores negativos^1^. Em vários casos, o único sintoma apresentado foi a dor torácica progressiva. Há ainda relatos de dispneia, sopro contínuo, frêmito torácico, edema doloroso paraesternal e até massa supraclavicular ou intercostal. Cerca de 37% dos casos manifestam-se com rotura do aneurisma, causando hemotórax maciço e risco potencial de óbito^1^. A letalidade desses aneurismas pode ser explicada pela localização dentro da cavidade torácica. A pressão intratorácica subatmosférica, o movimento dinâmico da parede torácica e a relativa falta de tecido de suporte adjacente criam um ambiente ideal para o crescimento do aneurisma e para sangramento volumoso em caso de ruptura^11^. Além disso, a expansão do aneurisma ou hematoma contido pode levar a compressão e paralisa do nervo frênico^11^. Esses são os principais motivos para a indicação cirúrgica dos AATIs^1^.

A descrição exata do tamanho e a localização anatômica do AATI é crucial para o planejamento cirúrgico. Das várias modalidades de imagem não invasivas, a angiografia por tomografia *multislice* é o exame de escolha para o diagnóstico. Com esse exame, é possível visualizar o aneurisma de maneira muito detalhada, usando técnicas de pós-processamento com formatação multiplanar e análise de volume^11^. A parede de um aneurisma é geralmente lisa e bem delineada, com exceção do aneurisma micótico, no qual a parede pode estar mais espessa, irregular e mal definida. Além disso, permite visualização do vaso nutrício e dos vasos colaterais, importantes para o planejamento cirúrgico^14^.

Os AATIs verdadeiros são tão raros que existem informações limitadas sobre seu manejo e prognóstico. Até o momento, não há critérios de intervenção estabelecidos^3^, e a decisão de tratar os AATIs é baseada no tamanho, na presença de sintomas e no risco de ruptura^8^. A ruptura dos AATIs pode ser fatal, tendo em vista que o hemotórax com choque é a manifestação inicial mais comum^3^. As opções terapêuticas descritas na literatura são o reparo cirúrgico aberto e o tratamento endovascular com implante de stent e/ou embolização. No caso da ruptura, o tratamento depende da condição hemodinâmica do paciente.

Em pacientes instáveis após ruptura, a cirurgia aberta ainda é considerada o método de escolha^1^. A exploração cirúrgica inclui a remoção do hematoma, a ligadura cirúrgica do vaso e o empacotamento para obter hemostasia^15^. No entanto, a cirurgia é agressiva e a identificação da fonte do sangramento pode ser difícil, assim como a ligadura cirúrgica devido à natureza frágil do tecido vascular. Miura et al.^16^ relataram uma tentativa frustrada de identificar a causa do sangramento em um paciente com ruptura de aneurisma de artéria intercostal. O reparo cirúrgico aberto apresenta ainda outros riscos, como sangramento, infecção do sítio cirúrgico, lesão de estruturas adjacentes, riscos relacionados à anestesia, recuperação prolongada e maior tempo de internação hospitalar^8^^,^^14^. Assim, o tratamento endovascular vem se tornando uma opção terapêutica eficaz, menos invasiva e segura, mesmo para pacientes instáveis hemodinamicamente^17^.

Em pacientes hemodinamicamente estáveis, o tratamento de primeira linha é o endovascular, por ser um procedimento minimamente invasivo muito utilizado em idosos, pacientes graves, com distúrbios de coagulação, bem como condições especiais, como pacientes com síndromes de Marfan e Loeys-Dietz^11^^,^^13^. A embolização com molas é o tratamento de escolha para fístulas arteriovenosas e aneurismas menores devido à facilidade técnica que elas proporcionam em vasos tortuosos e em colos curtos^3^. Há ainda outros agentes utilizados para embolização que podem ser combinados na terapêutica com o objetivo de ocluir a porção proximal e distal do aneurisma para evitar a nutrição por colaterais, como polímeros e eventualmente colas^11^.

A exclusão do aneurisma por meio de stents recobertos é uma opção para determinados leitos vasculares e alguns trabalhos demonstraram ser um tratamento alternativo factível para AATI^18^. Alhawasli et al.^19^ relataram uma exclusão bilateral bem-sucedida de AATI empregando stents sequenciais recobertos em um paciente com síndrome de Marfan. Alguns autores acreditam que há benefício em preservar a perviedade da artéria torácica interna, principalmente nos pacientes com alto risco cardiovascular, tendo em vista uma eventual necessidade futura de revascularização do miocárdio^1^.

O refluxo por colaterais levando ao crescimento do aneurisma, bem como o ressangramento após embolização, foram identificados como complicações de procedimentos endovasculares^11^. Entretanto, a taxa de sucesso dos AATIs embolizados é de 94,3%^11^. Dessa forma, as técnicas endovasculares oferecem uma alternativa minimamente invasiva, eficaz e segura, sendo atualmente o tratamento de escolha para os AATIs^14^.
